# Parenting quality in drug-addicted mothers in a therapeutic mother–child community: the contribution of attachment and personality assessment

**DOI:** 10.3389/fpsyg.2014.01009

**Published:** 2014-09-10

**Authors:** Francesca De Palo, Nicoletta Capra, Alessandra Simonelli, Silvia Salcuni, Daniela Di Riso

**Affiliations:** ^1^Department of Developmental and Social Psychology, University of PaduaPadua, Italy; ^2^Therapeutic Community “Casa Aurora” – Comunità di Venezia s.c.s., VeniceItaly

**Keywords:** adult attachment interview, psychiatric symptoms, mother–child relationship, therapeutic community, drug addiction

## Abstract

Growing evidence shows that attachment is a key risk factor for the diagnosis and treatment of clinical diseases in Axis I, such as drug addiction. Recent literature regarding attachment, psychiatric pathology, and drug addiction demonstrates that there is a clear prevalence of insecure attachment patterns in clinical and drug addicted subjects. Specifically, some authors emphasize that the anxious-insecure attachment pattern is prevalent among drug-addicted women with double diagnosis (Fonagy et al., 1996). The construct of attachment as a risk factor in clinical samples of drug-addicted mothers needs to be studied more in depth though. The present explorative study focused on the evaluation of parenting quality in a therapeutic mother–child community using attachment and personality assessment tools able to outline drug-addicted mothers’ profiles. This study involved 30 drug addicted mothers, inpatients of a therapeutic community (TC). Attachment representations were assessed via the Adult Attachment Interview; personality diagnosis and symptomatic profiles were performed using the Structured Clinical Interview of the DSM-IV (SCID-II) and the Symptom Check List-90-R (SCL-90-R), respectively. Both instruments were administered during the first six months of residence in a TC. Results confirmed the prevalence of insecure attachment representations (90%), with a high presence of U patterns, prevalently scored for dangerous and/or not protective experiences in infanthood. Very high values (>5) were found for some experience scales (i.e., neglect and rejection scales). Data also showed very low values (1–3) in metacognitive monitoring, coherence of transcript and coherence of mind scales. Patients’ different profiles (U vs. E vs. Ds) were linked to SCID-II diagnosis, providing insightful indications both for treatment planning and intervention on parenting functions and for deciding if to start foster care or adoption proceedings for children.

## INTRODUCTION

In Italy, pregnant women or women who deliver under the influence of addictive substances receive a warning from the Substance Addiction Treatment Services (Ser.T) and the Juvenile-Less-Protection Services (Social Services, Juvenile Court) with the aim to activate a protection protocol for the child’s conditions, since its birth or the very first months of life. Basing their perspective on the protection protocol for child’s conditions, health care services and Therapeutic Community’s (TCs) might improve their ability to define personalized mother–child dyad evaluation and treatment as soon as possible, in order to reduce risk of failure or not-useful therapeutic planning (Stocco et al., 2012). The general aim of this paper is underlined the importance of the integration of contributions devised by attachment and personality assessment, to delineate clinical “mothers’ profiles.” Starting from the individual’s affective-relational functioning, considering the quality of the state of mind in respect to attachment, and the clinical symptomatology, the definition of mothers’ profile highlighted early indicators of risks and/or protection of the parental functioning, as well as indicators about the treatment compliance and outcomes for these patients.

### THEORETICAL BACKGROUND

Attachment is a biological-based system with the goal of increasing protection from dangers and predation, comfort during times of stress, and social learning, through the manifestation of attachment-related behavior in infancy (e.g., clinging, crying, monitoring caregivers, and developing a preference for a few reliable caregivers; Davila et al., 2005). The process through which attachment patterns in childhood are stable overtime is given by the fact that early experiences with caregivers are transformed into internal mental representations of attachment during childhood and adolescence, the Internal Working Models (IWM; Bowlby, 1973, 1980). IWM are theorized as beliefs about the self and others from which rules are derived and used to guide behavior (Bowlby, 1973). IWM develop from repeated interactions with caregivers and their quality is linked to caretakers’ responsiveness during episodes of distress (Thompson and Raikes, 2003; Marvin and Britner, 2008). These internal representations are hypothesized to act as filters for future relationships and experiences, affecting behaviors by influencing quality and regulation/dysregulation of emotional experience (Carlson et al., 2004). Bowlby (1973, 1980) suggested that negative representations of the self or others are due to the influence of strategies for processing attachment-related thoughts and feelings which compromise realistic appraisals: in such situation, the child and the adult become more vulnerable to psychopathology.

Attachment theory emerged as a useful developmental model, with several implications for psychopathology and clinical treatments, and provided clinicians and researchers with a method to examine the impact of early experiences on later adjustment (Sroufe, 2005; Slade, 2008). Infants’ early attachment to their caregivers is considered a key developmental task which influences: (a) child’s representations both of self and others, (b) the individual’s strategies for processing attachment-related thoughts and feelings during the cycle of life (Dozier et al., 2008). Bowlby (1969, 1973, 1980), (c) the affective bonding between infants and their caregivers, and (d) the long-term effects of early attachment experiences on personality development, interpersonal functioning, and psychopathology in terms of behavioral systems (George et al., unpublished manuscript; Fonagy and Target, 1997).

Attachment insecurity, although originally defined as an adaptive set of strategies designed to manage distress (George and West, 2012), increases vulnerability to psychopathology. Moreover, recent findings in the literature about attachment and psychiatric pathology demonstrate more and more that there is a clear prevalence of insecure attachment patterns in clinical subjects as assessed by the Adult Attachment Interview (AAI; George et al., unpublished manuscript; Hesse, 2008). The classical meta-analytic study by Fonagy et al. (1996) as well as the meta-analytic findings in Bakermans-Kranenburg et al. (2008) highlighted the prevalence of insecure attachment, when the three-way classification system was used, and of the Unresolved with respect to loss and/or trauma (U) attachment, when the four-way classification system was used.

Recent studies linked attachment constructs with psychopathology, including depression, anxiety, eating disorders, and personality disorders. In respects with personality disorders, several studies investigated the association between attachment, assessed by the AAI, and incidence of Borderline Personality Disorder (BPD) in clinical samples (Davila et al., 2005; Levy, 2005; Dozier et al., 2008); Fonagy et al. (1996), using the three-way classification system, found that 75% of the subjects diagnosed with a BPD had a Preoccupied (E) state of mind and half of them belonged to the rarely used “fearful preoccupied with respect to trauma” (E3) sub-group. The same results were obtained by Patrick et al. (1994) and by Rosenstein and Horowitz (1996): authors found that the majority of BPD patients (64%) were classified E at the AAI. Preoccupied attachment often co-occurs with unresolved status. Not surprisingly, when the four-way classification system was used, 89% (Fonagy et al., 1996) and 75% (Patrick et al., 1994) of BPD patients were classified as Unresolved with respect to a Loss and/or a Trauma (U). Brennan and Shaver (1998) described persons with a fearful attachment as the most troubled ones, with a high prevalence of distortion of reality or negativity about others (Allen et al., 1998). Within the attachment theory framework, BPD adults could be considered the “product” of early dysfunctional parent–child relationships: BP subjects, with their pervasive needs for approval and acceptance and their difficulties in emotion regulation (Brennan and Shaver, 1998), generally grew up in a condition of inconsistent and unpredictable parents’ responses; these parental attitudes induced an increase of attachment system activation, in spite of the explorative and assertive systems (George and West, 2012), leading to the development of an insecure self-image, an unreliable others’ image, deficits in empathic attunement, social and relational maladjustment and affect dysregulation in children (Shorey and Snyder, 2006).

Many researchers found that BPD diagnosis (Axis II) is highly correlated with Substance Dependence Disorder (SDD, Axis I), especially for women (Gunderson, 2001; Gunderson et al., 2010). Women characterized by SDD and BPD are socially unsuccessful, emotionally instable and explosive, more likely to incur acting out and episodic psychotic experience, including intra-psychic experiences of splitting, identity diffusion, projective identification (Kernberg, 1977; Gunderson et al., 1981). To our knowledge, even if the correlation between BPD and insecure attachment was clearly demonstrated (see Fonagy et al., 1996; Ward et al., 2001 for a review), so far there is a paucity of research providing empirical support to the association between attachment and SDD. Moreover, studies conducted using the AAI (George et al., unpublished manuscript) suffered from a limited sample size or provided inconsistent results (Caspers et al., 2006). To the extent that substance abuse can be a self-medicating strategy intended to mitigate or protect against distress (Khantzian, 1985), insecure individuals may be more likely to abuse substances as a way of regulating negative feelings. For example, Rosenstein and Horowitz (1996) found a higher rate of substance abuse among adolescents classified as dismissing than among the ones classified as Preoccupied. Consistent with Rosenstein and Horowitz’s (1996) findings, Allen et al. (1996) found a significant positive association between problematic substance use and those AAI scales which are usually attributed to a dismissing state of mind (e.g., derogation of caretakers), while a negative association was found between problematic substance use and those scales which are usually associated with Preoccupied attachment (e.g., involving anger). However, such studies failed to find a significant overall effect of attachment category on substance abuse. Similarly, one study found that a scale associated with dismissing attachment (i.e., derogation of attachment experiences) was related to current hard drug use among previously hospitalized adults (Allen et al., 1996). Support for these results came from Mickelson et al. (1997), who found significantly higher rates of alcohol and drug abuse among dismissing individuals when compared to Secure or Preoccupied subjects. Further support came from Borelli et al. (2010) who examined attachment-related differences among the four AAI groups and predicted that compared to secure women, dismissing, and unresolved women would be more likely to have a history of substance abuse and to have been incarcerated for drug related crimes. They also predicted that these drug-related variables would be associated with greater derogation of attachment and greater lack of resolution of loss or trauma as assessed by AAI. In line with these results, Ward et al. (2001) found a significant association between substance abuse and dismissing state of mind with respect to attachment. However, these data are in contrast with some authors who have emphasized that preoccupied attachment is the most frequent classification among drug-addicted women with double diagnosis (Fonagy et al., 1996). In contrary, when the four-way system of the AAI classification is considered, Fonagy et al. (1996) and Riggs and Jacobvitz’s (2002) studies showed that substance abuse is more common among individuals with Unresolved attachment classifications.

Although different clinical groups assessed by means of the AAI showed a high proportion of insecure and unresolved attachment patterns, a clear association between a specific psychiatric clinical diagnosis (based on Axis I and Axis II) and a peculiar attachment pattern was never found. Moreover, data did not lead to a specific attachment indication about SDD (Caspers et al., 2006): the authors explain this gap referring to many limitations connected to the samples, which compromise the applicability of research designs. In addition, when considering research designs, several other factors need to be mentioned, such as the number of variables taken into consideration and the type of data analyses. Last but not least, such studies often failed to address the issue of the presence in the sample of subjects with double diagnosis. These may also be time parents at the same (in particular mothers), showing a psychic condition that might be made even more problematic by the presence of a child and by the necessity to exert a parenting function.

### AIM

Many mother–child TCs are present on the whole national territory: they greatly differ from each other in terms of constituent aspects, which have recently been included into a complex and articulated regulation. This new regulation leaves space to autonomous choice for regional organization and definition (available places, internal arrangement, monthly fee, etc.). In the first place, TCs give hospitality to drug-addicted women (already detoxified or on methadone therapy and followed by the Ser.T.) and their children, to whom a comprehensive rehabilitation path is offered, including parenting support.

As for the intervention methods, a combined treatment (i.e., for both parent and child together) is carried out on an intensive basis (the dyads are in residential care): TCs typically offer a therapeutic rehabilitation program, which is centered on the individual–parent–child system taken as a whole (Meisels et al., 1993). Admitting the mother–child dyad into a TC means, on the one hand, the possibility of guaranteeing an adequate intervention for the adult while providing a protective environment for the child; on the other hand, it allows to monitor mother’s mothering and parenting capacities of taking care of her child autonomously, and/or to learn how to do it in an adequate manner. This implies the risk for these mothers to be evaluated as incapable of caring for their children and, consequently, the risk of being separated from them (Stevens et al., 1989; National Institute on Drug Abuse, 1996), when a good outcomes in treatment programs will not reached.

The aim of the present paper was to investigate, in a very homogeneous diagnostic sample, the possible associations between the quality of state of mind in respect with attachment and psychopathological characteristics of SDD (Axis I) and BPD (Axis II) in mothers hosted with their children in an Italian TC, in order to suggest specific treatment methods able to obtain a more positive outcome with these addicted women, also taking into account the relationship with their children.

All mothers in our sample were clustered into four profiles, characterized by the same clinical diagnosis (SDD and BPD), but with differences in affective and relational attachment patterns (F – Ds – E – U) and in levels of symptomatology (SCL-90-R positive subscales): *Warm*, *Cold*, *Hot*, and *Unpredictable* mothers’ profiles. Data were used in a clinical qualitative approach to delineate groups’ affective-relational functioning, considering the quality of the state of mind in respect with attachment, and clinical symptomatology. Furthermore, in an intervention stance (Wallin, 2007), the paper aimed to individuate early indicators of risks and/or protection of the parental functioning, as well as indicators about treatment compliance and outcomes.

## MATERIALS AND METHODS

### PARTICIPANTS

This study examined 30 mothers (*M* = 30 years; SD = 6.5)^1^. In our sample, the 43.3% of mothers was sent to TCs according to a court decree, whereas the remaining participants were volunteers. The main aim of accessing treatment was the detoxification from substances, in order to be able to take care of their children (i.e., rehabilitation path to parenting support).

Regarding substance abuse history, all mothers during the 12 months period before entrance in the community, showed a pathological pattern of abuse or use of substances which led to a significant impairment or distress: 22 (73.3%) mothers used heroin, 5 (16.7%) cocaine, and the 3 (10.0%) alcohol. The mean age for the onset of the dependence was 17 years (SD = 4.6), whereas the intoxication period lasted on average 12 years (from 3 to 24). Participants reported that during that time they were affected by a persistent desire to get substances, never considering physical or psychological problems, which may be related to the dependence; they recognized how substance abuse deeply influenced their type of social, occupational, and recreational activities.

Focusing on the medical history, 12 mothers (40%) in this sample reported drug-related pathologies: in particular, HIV (*N* = 1), HBV (*N* = 1), and HCV (*N* = 10). Moreover, the majority of them was subjected to a pharmacological treatment: 21 mothers (70%) out of 30 used opioids (Methadone, Subotex, Naltrexone) to substitute or reduce addiction; 8 mothers (27%) daily had antipsychotics (Seroquel, Talofen, Inpromen); 6 (20%) had benzodiazepines, only 2 (6%) out of 30 had anti-depressives. All mothers may have a set of diverse symptoms at the same time and for this reason they could follow more than one pharmacological treatment. Taking into consideration the social and environmental context, several aspects were analyzed: first of all, regarding education and work, 21 participants (70%) ended the middle school and 9 (30%) others attended a professional school. Before the access in service, 22 (73.3%) mothers were unemployed and the rest were dependent employees. All mothers reported past trauma experiences: 21 (70%) experienced maltreatments (eight physically, nine psychologically, and four sexually); 18 (60%) participants suffered from an unresolved loss, 11 (37%) attempted suicide, 9 (30%) had an overdose experience, and 10 (34%) were engaged in prostitution acts. As mentioned above, all participants were mothers who, generally, had pregnancy and parenting difficulties with consequences on child’s development: 21 (70%) of both mothers and their partners used drugs during pregnancy, and 9 (30%) of children were born with Newborn Abstinence Syndrome.

### PROCEDURE AND INSTRUMENTS

Therapeutic Community for drug-addicted women and their children offers residential care to the mother–child dyad and provides a comprehensive rehabilitation program, which takes place during a two-year stay^2^. During the first month in TC, participants underwent an assessment phase: specifically, the central issues were substance addiction and medical history, social-environmental influences and other psychiatric symptoms and adult attachment patterns (Stocco et al., 2012). All participants were administered a set of measures.

– *The Structured Clinical Interview for DSM-III-R* (SCID-II; First et al., 1997; Italian version by Mazzi et al., 2003) allows diagnostic evaluations of a potential personality disorder, such as the ones included on Axis II of DSM-IV, passive-aggressive, and depressive disorders (Appendix B of DSM-IV), and unspecified personality disorder.
– *The SCL-90-R* (Derogatis, 1983; Italian version by Sarno et al., 2011) was used in order to evaluate psychological problems and symptoms of psychopathology. This is a relatively brief self-report questionnaire published by the Clinical Assessment division of the Pearson Assessment & Information group. It is designed to evaluate a broad range of psychological problems and symptoms of psychopathology. It consists of 90 items and takes 12–15 min to be administered, yielding nine scores along primary symptom dimensions and three scores among global distress indices. The primary assessed symptom dimensions are somatization, obsessive-compulsive, interpersonal sensitivity, depression, anxiety, hostility, phobic anxiety, paranoid ideation, psychoticism, and a category of “additional items” which helps clinicians assessing other clients’ symptoms. The three indices are Global Severity Index (GSI), considered to be a more sensitive single quantitative indicator, concerning respondent’s psychological distress status; Positive Symptom Distress Index, considered to be an intensity measure, which may also provide information about respondent’s distress style; Positive Symptom Total, which reveals the number of symptoms that the respondent has endorsed to any degree. Particularly, criteria to interpret the GSI score are: with *T* < 55 subjects’ general level reported is normative, 55 ≤ *T* ≤ 65 subject reports from moderate to high level of disease; *T*> 65 subject reports a level of disease over the clinical cut-off.
– *The AAI* (George et al., unpublished manuscript). This semi-structured interview aims to elicit information concerning an individual’s current representation of his or her childhood experiences with the attachment figures. The interview consists of questions through which the participant is asked to recall and to reflect upon memories related to his/her attachment experiences with his/her caregivers during childhood. The AAI coding system is divided in two phases. First, the protocol is coded according to the Scales of Subjective Experience (Loving, Push to achieve, Rejection, Neglect, Role reversing) and Scales of the State of Mind (Coherence of transcript, Metacognitive monitoring; Idealization; Lack of memory; Passivity; Anger; Derogation; Coherence of mind; Fear of Loss; Unresolved Loss; Unresolved trauma) with respect to attachment, relying on both form and content. Each of these scales may receive a score based on a 1–9 Likert Scale: levels score were considered Low (<4), Mild (4–7) and High (>7) (Main et al., unpublished manuscript). Secondly, interviews are considered as a whole and classified into the adult attachment categories: secure (F), Dismissing (Ds), Entangled-Preoccupied (E), Unresolved with respect to a Loss and/or a Trauma (U). Interviews are audio taped and transcribed. Two raters, who were unfamiliar with the sample and who had no access to demographic and psychiatric information, rated the interviews independently. Both raters had been trained in conducting this coding, and had substantial experience with this instrument. The AAI interviews are rated either in respect to evaluation scales and to the general attachment category. The inter-rater reliabilities of the scales used by the present raters were all higher than 0.80. The inter-rater reliabilities of raters in this study were consistent with values reported in the literature: 85% on major attachment classification (100% agreement on insecure vs. secure classification), and between 70 and 80% on sub-classification categories (Bakermans-Kranenburg and van IJzendoorn, 1993).

## RESULTS

Regarding the personality structure evaluated according to DSM-IV-TR (American Psychiatric Association [APA], 2000), all participants reached SDD criteria in Axes I, BPD criteria in axes II according to the SCID-II (First et al., 1997), general medical conditions at risk (Axes III), and, psycho-social and environmental problems (Axes IV).

Focusing on SCL-90-R, a peculiar finding is that neither the means of subscales nor the means of GSI score ever overcame *T* = 65 (i.e., cut-off for the clinical range). According to our perspective, this could be caused by a multiplicity of factors, which go beyond mothers’ psychopathology. Their self-reported symptomatology could highlight their tendency to “underscore” psychiatric symptoms partially, in order to prevent negative evaluations and its consequences in TCs: they tend to show themselves in a better way as a consequence of their accessing the community due to an assessment purpose in order to maintain their relationship with their children. Depression scale was a common factor in all profiles. Regarding the prevalence of depressive symptoms, we firstly need to consider that these mothers have just accessed a mandatory residential treatment, have just been separated from their own life-context. Secondly, all our mothers are very likely living a hard period of abstinence from substances, when they are experiencing parenting with responsibility toward their children, and they know health care community employers are due to assess their parenting abilities. For these reasons, the presence of depressive aspects will not be discussed in the following profiles, because they are considered clinical characteristics, which are common to all subjects, thus not particularly significant for the main purpose of this categorization.

Furthermore, another common factor in all mothers is the positive Psychoticism scale: we hypothesize that aspects of hostility, sensation seeking, and impulsivity may be connected to the diagnosis of BPD.

As shown in **Table 1**, there was a highly significant proportion of insecure attachment categories in the three-way distribution of attachment patterns [*F* = 4 (13,3%); DS = 14 (46,7%); *E* = 12 (40%)] as well as in the four-way distribution of attachment patterns [*F* = 4 (13,3%); DS = 9 (30%); *E* = 7 (23,3%); U/CC = 10 (33.3%)]: these data seemed in line with previous literature about AAI distribution of attachment patterns in clinical groups, confirming a prevalence of insecure models, independently from the considered diagnostic category (Bakermans-Kranenburg et al., 2008). However, when considering the 3-categories distribution, overlying values of DS and E attachment patterns emerged, consistently with data obtained from groups of subjects with BPD and with SDD (Dozier et al., 2008). Considering a 4-category distribution, higher percentage of the U attachment pattern lead with the researches on subjects with substance dependence and double diagnosis (Fonagy et al., 1996).

**Table 1:** Mothers profiles in respect with attachment patterns and SCL-90-R subscales.

SDD-BPD mother profiles	Attachment pattern	AAI State of mind scales^1^	AAI Experience scales^2^	SCL-90-R Global symptom index^3^	SCL-90-R Subscales^4^ 55 ≤ T ≤ 65
WARM	Secure (F) *N* = 4	Coherence mind** Coherence Tr** Metacognitive monitoring** Idealization** Lack of memory* Passivity* Anger* Derogation* Fear of loss* Unresolved Loss* Unresolved trauma*	Loving** Push toachieve* Rejection* Neglect* Role reversing*	*T* = 51,25 Normal distress	Interpersonal Sensitivity Psychoticism Depression Anxiety Paranoid ideation
COLD	Dismissing (DS) *N* = 9	Coherence mind* Coherence Tr* Metacognitive monitoring* Idealization** Lack of memory** Derogation** Anger* Passivity* Fear of loss* Unresolved loss* Unresolved trauma*	Loving* Rejection* Neglect** Role reversing* Push to achieve*	*T* = 53,56 Normal distress	Depression Psychoticism Obsessive–compulsive Symptoms Hostility
HOT	Preoccupied (E) *N* = 7	Coherence mind* Coherence Tr* Metacognitive monitoring* Passivity** Idealization* Anger** Lack of memory* Derogation* Fear of loss* Unresolved loss * Unresolved trauma*	Loving** Role reversing** Rejection* Neglet* Push to achieve*	*T* = 58,29 Distress from moderate to high	Psychoticism Depression Anxiety Interpersonal Sensitivity Somatization Paranoid ideation
UNPREDICTABLE	Unresolved (U/CC) *N* = 10	Coherence mind* Coherence Tr* Metacognitive monitoring* Passivity** Idealization** Anger** Derogation* Lock of memory* Fear of loss* Unresolved loss* Unresolved trauma**	Loving* Neglect** Rejection** Role reversing* Push to achieve*	*T* = 61,30 Distress from moderate to high	Psychoticism Paranoid ideation Interpersonal Sensitivity Hostility Anxiety Obsessive–compulsive Symptoms Depression Somatization

^1^The State of Mind Scales end the Experience Scales means score ranges: *(<4), **(4–7), ***(>7) (Adult Attachment scoring and classification Systems, Main et al., unpublished manuscript).

^2^The State of Mind Scales end the Experience Scales means score ranges: *(<4), **(4–7), ***(>7) (Adult Attachment scoring and classification Systems, Main et al., unpublished manuscript).

*^3^Criteria for the interpretation of scores: *T*< 55 general level of distress in the normative range, 55 ≤*T* ≤ 65 subject reports from moderate to high general level of distress; *T*> 65 subject reports a level of distress in the clinical range.*

*^4^Scales are decreasingly presented, from the one with the highest score to the one with the lowest.*

From a qualitative clinical point of view, we define 4 “Mothers’ profiles” (*Warm*, *Cold*, *Hot*, and *Unpredictable profiles*) starting from attachment patterns (F – Ds – E – U, respectively), also evaluating AAI subscales, and their associations with level of psychiatric symptomatology (SCL-90-R subscales): indicators of risks or protection of parental functioning and of treatment compliance and outcome are discussed.

*Warm profile* characterized mothers with SDD and BPD, Secure pattern of attachment and few symptoms. In this profile, as regards psychopathologic features and considering only subscales in the range between 55 and 65, five scales out of nine emerge as significantly relevant. More specifically, interpersonal sensitivity shows the highest score, followed by psychoticism, depression, anxiety, and paranoid ideation, decreasingly. Overall, the GSI score highlights a normative amount of general distress. Subjects presented primary experiences with attachment figures characterized by loving relationship. Coherence of mind and metacognitive monitoring emerged as aspects of principal functioning related to their past attachment experiences. Furthermore, the mild use of defensive strategies, such as idealization, lack of memory, and passivity, remains. Generally, some affective-relational aspects have been observed in Warm profile mothers: even though they experienced both positive and negative relational experiences in infanthood, they are able to depict, describe, and meditate on the complexity they lived.

These mothers may be considered in some ways as “earned secure” subjects, that means those people who show secure states of mind in respect to attachment in adulthood, even if they did not live optimal experiences in childhood (George et al., unpublished manuscript). In other words, they processed a mental re-organization: from an unsecure attachment pattern in childhood, to a more balanced and judicious mental organization in adulthood. These women tend to be particularly acute and meditative, presenting a good reflection functioning, which enables them to integrate difficult memories, which are often threatening and discrepant. They are usually able to describe either the way in which their early experiences had contributed to make themselves the women they are, or the way in which their parents’ parenting toward them had influenced their own parental functioning toward their children.

An example from an AAI is presented below:

AAI Question: Are there aspects of these early experiences in childhood that you think they might have been a disadvantage or that have obstructed your development?

Answer: yes, yes, because. They many times indeed. They always knew what was the best for me, and this took away from me the chance to choose by myself, didn’t it? And…. Because you say: “no!, for me this is not right, I think something else, I want to do something else,” and then I did it, with no support though, and so it is easier for someone to make a mistake, because you start saying: “but I am doing something against… with no blessing, and.. so it is more difficult,” so, they supported less and less my real personality, they have always believed.. in a good way. But to know what was right for me”

With the increase of reflecting and auto-observation functioning, defensive mechanisms typical of BPD, such as defensive exclusion of bad memories (Lack of memories; George and West, 2012) and use of idealization, diminished. Being capable of reflecting on their own relationships allowed them to regain their contradicted memories, giving a more coherent picture of life experiences. This goes with a higher comprehension of their own actual problems and with a higher acceptation of a possible therapy. Overall, *Warm profile* mothers experienced both loving and not protective experiences in childhood; they have a state of mind able to use both defensive strategies (as idealization, lack of memory, and passivity) and, at the same time, reflective strategies (as, good ability in mentalization and coherence of mind). The latter allows modulating dysfunctional characteristic of the BPD. However, difficulties linked to interpersonal sensitivity, psychoticism, anxiety, and paranoid idealization persisted, in particular when left alone in stressful situations. Even when considering SDD, mothers belonging to the *Warm profile* seemed to be able to talk about drug abuse experiences, understanding the underneath needs that bring them to drugs assumption, and diminishing the necessity of drug consumption. They are not safe from the risk of relapse into drug addiction, but, overtime, they learn how to interpret that need.

A secure autonomous state of mind with respect to attachment provides parents with flexibility of thinking and ability to regulate their emotions, allowing them to respond with sensitivity and empathy to their infants’ distress. On the other hand, women with BPD are characterized by intrusive insensitivity, based on ratings of their speech and behavior, by displaying deregulated affective communication toward their infants, including critical and intrusive behaviors as well as the rejection they were subjected in childhood (Kiel et al., 2011; Stepp et al., 2012). The presence of both these patterns in the *Warm profile* resulted in a complex parenting style: they may express a responsive behavior, associated to an underneath state of fear, tension, or anxiety; then, being able to reflect on their own behaviors, they may solve this tension, detaching themselves from infants. These women may exhibit tension when intimately approaching their children, because proximity makes them vulnerable. However, this difficulty is only a feeble physical detachment, rather than an active and negative refuse of communicating with the infant.

Talking about compliance and therapeutic alliance during treatment, secure subjects are predisposed to consider the therapist as a secure base, using it to “tune” an attachment paradigm both in an operative and valuable way. Satterfield and Lyddon (1995) confirmed this hypothesis analyzing the therapeutic alliance: they observed that patients with a secure attachment style had a propensity to establish bonds and negotiate treatment goals with their mental health consultants. As mentioned above, secure attached patients are generally able to reveal their thoughts and their feelings, recognizing that their own relational models influence their life experiences. Because of the possibility to establish a therapeutic alliance with them, treatment toward *Warm profile* mothers had the purpose of remodeling the personality asset and of improving their global functioning, principally working mostly on aspects related on identity confusion, chronic feelings of emptiness and relational instability (Wallin, 2007).

Mothers belonging to the *Warm profile*, in line with their baggage of reflective abilities, resulted to be able to reach a significant therapeutic alliance and therapeutic change. However, a larger awareness of their own mistakes causes them more suffering. In particular, metacognition strategies lead to strong feelings of responsibility for what has been done in the past, and for being in TC at present. Thus, a good treatment program for these mothers would need to help them to forgive themselves for their faults and to believe in their own abilities. Awareness about the complexity of their life experiences and capacity of metacognitive monitoring permits them to conclude the therapeutic path remaining together with their children, in spite of their BPD dysregulation pattern. Besides therapeutic moments of reflection that would help patients to face their past history of childhood and the connection between this and caregiving toward their kids, therapists directly intervene on this relationship, providing educative strategies to enhance the quality of it in everyday life. Strategies that would allow mothers to comprehend positive and negative aspects of the relation, but, above all, to strengthen mechanisms of repairing. Providing a new baggage of abilities to repair relational ruptures and the elaboration of traumatic experiences allow these women to re-acquire their paternal responsibility. These women are able to understand their limits in parenting skills and capacities, learn how to handle them, applying new strategies of caring, then their outcome could be rather positive. These mothers maintain the relationship with their children more or less autonomously even after the end of the therapeutic pathway.

An example taken from an AAI of this women’s modality to take care of their children is presented below:

AAI Question: Why do you think your parents behaved like you have described to me during your childhood?

Answer: Eh, surely because It was what they were subjected to in their childhood…yes, and then also because of the moment they were living in, so the moment between them and I believe it was fundamental to define in that moment their actions, and indeed this scares me thinking about Mark, I wouldn’t being in this situation because I know how much it is important to define the rule in which you - as a parent - have to be in the formation… Because it’s not that what you teach with words, but through the things people listen and see you are doing, so the proper example, so if you are peaceful, if you are... in the most possible way peaceful, in the most possible way quiet, what you transmit to a child, if you may have problems with your husband… so those are significant things that you don’t understand when you are experiencing them, no? And this scares me a lot.

The *Cold profile* characterized mothers with SDD and BPD, Dismissing pattern of attachment and, regarding psychopathological features, four subscales out of nine are relevant: depression presents the highest score, followed by psychoticism, obsessive-compulsive symptoms and hostility, decreasingly. Overall, the GSI underlines a normal level of distress. Subjects have primary experiences with attachment figures characterized by rejection and neglect. Regarding those characteristics of the state of mind, idealization, lack of memory, and derogation emerge as aspects of functioning related to their past attachment experiences. Not receiving comfort and experiencing refusals from attachment behaviors, a double defense mechanisms of affectivity are used: parents’ qualities are split between positive and negative, but only the firsts are recognized, whereas the seconds are inhibited; through the second mechanism, instead, they remember bad experiences, but they excuse their parents for them. In this case, subjects take their parents’ point of view, and, in line with this, they base their behavior on cognitive expectations and deny their attachment needs and feelings. An example from an AAI is presented below:

AAI Question: Have you ever felt worried or frightened when you were child?

Answer: Not frightened, but worried…but I don’t remember exactly why probably because everything was going so bad and I was trying to let it roll right off my back, I didn’t give any kind of satisfactions, even when my mom battered me I remember it hurt me but I forced myself not to cry

*Cold profile* mothers use thoughts and behaviors with the aim to defensively exclude painful feelings from awareness: from this perspective obsessive-compulsive symptoms focused on control of emotion, and BPD defense mechanisms are characterized by idealization and devaluation of self and others, which consistent with the constraint of affectivity and control.

In respect to the SDD, these women seem to deny their dependence from substances: as in the original attachment bond, they oscillate from an over-evaluation of their own abilities (“to not need it”), to endorsing downfall (“I can not live without it”).

Mothers belonging to the *Cold profile* exhibited controlled and dismissing engagement toward their children, showing high levels of push to achieve and perfectionism, low levels of attention to feelings and emotions, scarce attention to physical contact and loving attitude. In particular, maladaptive parenting behaviors may emerge in response to children’s emotional distress (Kim et al., 2009), interfering with their ability to respond adaptively to this distress, with negative emotional reactions (Leerkes and Crockenberg, 2009). Furthermore, *Cold profile* mothers may have increasing difficulty inhibiting insensitive behavior and/or displaying sensitive behavior as their infants’ distress persists. In this case, mothers are more likely to be characterized as intrusively insensitive, based on ratings of their speech and behavior, displaying dysregulated affective communication toward their infants, including critical and intrusive behaviors as well as the same rejection they were subjected to in childhood (Crandell et al., 2003; Hobson et al., 2005; Newman et al., 2007).

As regards compliance and therapeutic alliance during treatment, *Cold profile* mothers showed difficulties in establishing a healing relationship: they tended to express a strong message of autonomy and independency, and to deny needs to be supported even to the therapist. This kind of dismissing patients diminished or ignored intimacy and attachment values (Holmes, 1996), and tended to use the supportive therapeutic secure base (Dozier et al., 2008) to talk about others but not about their own self or themes, using intellectualization and taking distance from a real chance of re-elaboration (Holmes, 1998). According to Wallin (2007), therapists must be very attentive in order to take narrow affective signals; although dismissing patients are reluctant to express their own feelings, even if not on purpose they evocate and provoke reactions in the therapist. Therapists, highlighting their own feelings toward the treatment, may help patients to integrate their owns, even if dissociated or denied. In order to reach a good outcome, treatment might “translate empathy in words” (Wallin, 2007), reducing patients’ fear and their idea about therapist’s controlling and refusing behavior, with all possible misunderstandings. In this way, these mothers may be supported during the elaboration of their carelessness and refusal experiences, acquiring the ability to trust others in the therapeutic-relational context, asking and accepting necessary support. Therapists should provide them suggestions and behavioral strategies mainly functional, in order to enhance the interaction with children in everyday life. Specifically, therapist could initially function as a sort of model for handling interactions and for managing the tuning with the child. Successively, their abilities to interiorize could take to a gradual relational autonomy, that could permit them to interact with their child adequately.

Dismissing parenting characteristics might be considered not completely dysfunctional, as far as they show up as stable modalities of taking care of children, reassuring functional basic care; on the other side, the therapy role as secure base may enhance the access to these women’s affective sphere, increasing the possibility that they ask for help when child’s requests make it necessary. In this direction, *Cold profile* mothers’ prognosis might be better observed later in time, when they become able to do autonomously their own parental functioning, benefiting from the support of social and familiar networks in their life context. Indeed, mothers included in this profile may aspire to a total autonomy or, in case they are not able to overtake their dysfunctional patterns, maintain a central role of caring in their children’s life, sustained by a familiar context of support.

An example from an AAI of this women’s modality to take care of their children is presented below:

AAI Question: Thinking about how you talked, do you think there is something in particular that you have learned from your experience in childhood? I mean, what do you think you have got or understood, given the kind of infancy you had?

Answer: yes there would be that I will not do the same mistakes my mom did with me.. well it is not fucking true because sometimes things come up to mind so automatically that you can not even realize that, sometimes I should be sweeter to her but it is not automatic, fortunately I am here and there is someone who tells me that… I can learn…

*Hot profile* characterizes mothers with SDD and BPD, Preoccupied pattern of attachment and significant symptoms in six of the nine subscales of the SCL-90-R: the highest score is shown for psychoticism, then, decreasingly, depression, anxiety, interpersonal sensitivity, somatization, and paranoid ideation are significantly relevant. The GSI displays a general level of distress. Subjects included in this profile reported primary experiences with attachment figures characterized by the alternation of functional loving and role reversing behaviors; passivity and involving anger emerged at present in remembering their early attachment experiences.

Considering an affective-relational point of view, *Hot profile* mothers exhibited the inability to overpass their past history, and they still remained strongly linked to or worried about their own early attachment experiences. Affective status from their past influences actual narrative and thinking processes, making them vague and passive, creating confusion between perceptions of past, present and future. Self-concept appears as still trapped and involved in early negative relationships, which have not yet been elaborated. As a consequence, these women do not seem to be able to separate themselves from their own family identity, or from the inadequate experiences they lived (George et al., unpublished manuscript). Attachment experiences were lived as contradictory and incoherent (Shorey and Snyder, 2006): these women had partially positive experiences of functional loving care, alternated within adequate proximity situations and role reversing, in which parent “from taking care of someone, becomes the one who is taken care of” (George et al., unpublished manuscript). These experiences led to highly ambivalent IWM (Bowlby, 1973) in respect to self and others, defensively characterized by passivity, anger and cognitive disconnection (George and West, 2012) where no elaboration of thought is allowed.

AAI Question: Do you remember an episode, a memory that can explain why you think this relationship was confusing?

Answer: Ah because it could seem nearly that she adored me in a moment that she...would be happy and less after I don’t know what for how but all the contrary, that I was a bother that she was not happy about me... This confuses.. because one moment it was one way, and after another…. Even if it was laughing and joking about something and for one word she didn’t appreciate… so, as you were laughing as you would have found yourself crying less after. Needed ehm like it needed just a little thing, it needed nothing for her to being annoyed…

The example above highlights how *Hot profile* mothers’ states of mind are bound to an affective and relational dysregulation. This general distress status is manifested primarily through symptomatic aspects of psychoticism, anxiety, and interpersonal sensitivity. Given the strong negative feelings and the emotional over-involvement, substance abuse, and consumption seemed to be external mechanisms of regulation, which are activated when subjects experience uncontrollable and unmanageable painful emotions (Khantzian, 1985).

All these elements provided indications about *Hot profile* mothers’ parenting dysfunctional model. These mothers displayed disrupted affective communications, characterized by intrusive and insensitive behaviors, similar to that has been observed in studies with BPD samples (Hobson et al., 2009). In the relationship with their children they appear over-involved and over-stimulating; an active but not synchronized pattern of behaviors emerged (George and West, 2012). Furthermore, they expressed difficulties in tuning with their children positive affects, in line with studies conducted both on mothers with preoccupied attachment pattern (Haft and Slade, 1989; Deoliveira et al., 2005) and on BPD mothers (Crandell et al., 2003; Newman et al., 2007). In particular, these women reported difficulties in sharing expressions of infant’s happiness, linked to infant’s aspects of autonomy. Additionally, these mothers may more likely engage in role confusion with their children, and encouraging them to assume the parent or friend role.

In psychotherapy, *Hot profile* mothers are able to use the therapist as a secure base for affective and emotional support, but, similarly to patients with a preoccupied attachment patterns, they present difficulties in exploring and experimenting new relational possibilities (Obegi, 2008). On the contrary, differently from women’s of the previous profile, these patients may exaggerate their feelings, thoughts and physical disease, in order to get others’ attention and support, showing the desire to access the therapeutic too promptly. This approach to treatment could lead to endless programs, without obtaining any true change: these mothers appeared able to explore their own feelings in the therapeutic room, but they could not do the same in their real life context, precluding themselves the possibility to live new life experiences.

According to Wallin (2007), these patients might be helped reaching an emotional balance and reinforcing their own self-esteem. The therapeutic relationship should provide them with new alternative strategies, with the aim to disable the over-activation that they commonly use. For these reasons, therapeutic programs might be planned to establish a stable and very modulated relationship, in which the patient may count on the therapist’s emotional availability: the therapist might represent a sort of regulative and holding figure. *Hot profile* mothers tended to create a strong affective contact with their children. However, they could cause dysfunctional outcomes for infants’ growth, due to over-involvement, maladjusted emotional functioning, and to the great difficulties in handling child’s natural drive to separation and autonomy. If not healed, these mothers may be really dysfunctional for their children’s growth. A possible interactive intervention for these mothers led by the therapist regards the holding (building limits) of intrusive behaviors and the affective over-activation, which grow in contact with the child. Rarely, or with many difficulties, these mothers are able to interiorize these new relational strategies provided by the therapist: they are also predisposed to trust on and imitate it, using those strategies that these women interpret as more effective for the quality of the relationship with the child and the latter’s well-being. When dealing with extremely serious cases, it is fundamental to keep in mind that dividing the dyad over the first year of child’s age would be really dangerous since at that time the bond is so intense that, if broken, it would have more negative than positive effects.

In light of this, therapeutic outcomes include, on a side, a partial sharing of the parental responsibility with a “third” relational figure, as the foster home. This would be seen as an effective result, given the severity of these patients’ pathology: indeed, children can maintain the bond with their mothers, not being affected by their pathological oscillations. At the same time, foster home would guarantee the constancy of caring, protecting children’s development. On the other side, there are some situations in which, through distressing paths, women become aware of the chronicity of their pathology, taking them to choose to give their children up to adoption. Paradoxically, even these outcomes, if thought in light of child’s well-being, may be interpret as positive, because maternal support makes this painful ruptures more tolerable for children. These adoptions have better long-term outcomes than others overtime.

An example from an AAI of these women’s modality to be in relation with their children is presented below:

AAI Question: In general, how do you think experiences with your parents have influenced your personality as an adult?

Answer: Beh anyway even in her relationship, theirs, anyway I always said. I hope this does not happen to me… Yes no.. Well, because sometimes I felt guilty for being born, in the sense, talking to myself, yes and, if I were not there, maybe the two of them, they would not have been here fighting, all this kind of stuff, so then I thought, before having a child, in my life, I will think about it many times. And instead at the end, the exactly same thing happened to me, identical, in everything.. Either for age, because for example, even my parents have a twenty-year age difference and my boyfriend is twelve years older than me, so even for this, so for everything, seems that.. Everything I have ever said no I will never want something like that, [Smiles] And then instead, the contrary.

*Unpredictable profile* characterizes mothers with SDD and BPD, Unresolved and/or Cannot Classified patterns of attachment and significantly over the normative cut-off in eight SCL-90-R scales of psychiatric symptoms, with the only exclusion of the phobic anxiety scale. Overall, unpredictable mothers present a very severe symptomatic frame; psychoticism, specifically, reports a value (*T* = 65) which is just below the clinical range.

*Unpredictable profile* mothers’ affective-relational history is characterized by frequent maltreatment from caregivers, and/or by the presence of unresolved loss or trauma in childhood. Subjects present also primary experiences with attachment figures characterized prevalently by neglect and rejection. These early experiences have been stored in memory in an unelaborated way; segregated contents pop up automatically (George and West, 2012) in daily life, without any possibility of control, following free-associations with traumatic experiences experienced in childhood. When segregated traumatic contents emerged, these women manifest dissociative paths and disorganized thoughts, associated to absence of metacognition, and they consequently fall in a temporary condition of inability to employ control or to remedy on their thoughts.

AAI Question: In general, do you think that experiences with your parents, overall, might have influenced your personality as adult?

Answer: Yes, in a bad way. because I have lived in anxiety. I felt anxiety even when I was young, because it was not a peaceful environment, it was not a warm environment. It was an unpredictable environment. So I still feel anxiety from there. And I’m full of fears and fragilities. Because anyway when they were still alive I felt untouchable, now that they are not still here I feel like I’m a tiny point, even staying in, whatever, I have always thought that if they would be still alive probably I would not have been here. They would have done anything to figure things out. That they had always demonstrated to me, solving issue. Instead I know exactly more I feel alone, I don’t know, and I have to make anything by myself. And I have to struggle, it is so hard for me…I can cope with this…ever…”

For these women, the attachment disorganization entails a multiple and separated representation of the self and the attachment figure leading to a metacognitive deficit that makes emotion regulation extremely difficult. These features interact with the most significant clinical aspects of the BPD (i.e., impulsivity, oscillations between idealization and devaluation of self and others, feeling of emptiness, unmotivated and intense anger, auto-damaging behaviors, unstable and intense affective relationships) and in this way they amplify each other in a reciprocal manner. Overall, *Unpredictable profile* mothers present a highly compromised portrait, with persistent and disabling symptomatology, unresolved state of mind (characterized by feeble metacognitive abilities and strong dissociative mechanisms of thought), weak regulation of emotional experience, and a fragmented and diffused self. In this perspective, substance abuse and consumption seemed an extreme attempt of containment, through dissociative mechanisms, in order to approach the border between life and death, and to hold traumatic memories and experiences. However, these acting behaviors, especially in this case, expose individuals to high risk situations (overdose or suicidal behaviors).

In line with previous results, being subjected to traumatic unresolved experiences constitutes a risky condition for the undertaking of caregiving behaviors. Main and Hesse (1990) have posited that subjects whose mental statuses are immerged in painful unresolved memories – as for instance, memories of loss, incidents, diseases, maltreatment and violence – live in a pervasive condition of fear, that facilitates the assumption of caregiving behaviors of the threatening/timorous dissociated type. These caregiving behaviors appear out of the blue and with no contextual anchorage (Hesse and Main, 2006). Developing a mental condition of dissociation related to traumatic or loss experiences that have not yet been resolved, might be displayed by the attachment figures via a specific difficulty paying attention to infant’s painful emotional status, blocking the ability to regulate and modulate painful memories.

Mothers who belong to the *Unpredictable profile* exhibit behavioral patterns characterized by unpredictable modalities of interaction, and by a deregulated affective communication toward their infants, including critical and intrusive behaviors, role confusion, and frightened/frightening behaviors. More specifically, their children seem to live in a paradox in which they look for help and protection from their mothers, who, due to their unresolved and distressing memories, are frightened and, in turn, frightening. As a consequence, infant understands this latent fear and, consequently, reacts to this getting frightened. Talking about treatment issues, these disorganized patients lack any stable attachment strategy, struggling for the establishment of the therapeutic alliance, which results to be highly swinging, full of continuous outbreaks, interruptions, and rapprochements. As Holmes (2001) highlighted, therapists must tolerate this and, if necessary, approach to patient avoiding the classical setting. The therapeutic process with these patients is very long, but extremely gratifying. Once established, the alliance will be subjected to tensions and ruptures, and reparations represent the crucial action of the therapeutic task. The patient needs to find coherence. With the majority of these patients, the relationship with the therapist is a relevant part of the therapy: in the unresolved attachment group, the therapeutic relationship represents the therapy itself (Wallin, 2007). The treatment core is the integration of memories and experiences. Therapists’ mission is to provide different experiences and models of interaction, to create a relationship in which the patient can feel really safe. This is quite hard because these patients, although their willingness to cure themselves from their wounds, are often forced unconsciously to rebuild the well-known relational IWM with the therapist, in which there is no hope or possible help. It is important to underline that *Unpredictable profile* mothers must not be supported and led in therapy just to make them relive feelings linked to their traumas, but rather they need to recall those experiences and translate them into words, naming emotions and feelings. When patient is able to travel across these experiences again without being once more traumatized, at that moment memories are melt and changed, turning an omnipresent trauma omnipresent into one circumscribed in a specific time and place (Wallin, 2007). Given that these mothers’ prevalent interactive strategy involves the unpredictability of behaviors and emotional reactions in the relationship with the child, the therapist’s primary function could be to attempt to limit this modality, even substituting itself with the mother herself in taking care of the child, completely. In fact, considering that the interiorization and comprehension abilities are highly compromised, it is difficult for these women to acquire proper and functional models of interactions with their kids in absence of figures who could constantly give them behavioral and affective patterns to imitate and reproduce in everyday life.

Because of a particularly compromised clinical frame and a very long therapeutic work, *Unpredictable profile* mothers do not always reach the hoped therapeutic results. Situation gets worse when treatment outcome is considered in relation to children: these mothers scarcely come out from the TC program with their children autonomously. In the best case, a good outcome consists of being able to help these women to understand their limits in parental functioning, building together alternative pathways for the child, such as foster care or even adoption. In many cases, drop out occurs making it impossible to build anything good neither for themselves nor for their children. In these cases, mothers might leave the community or abandon infants, and professionals: given the impossibility of finding any openness in women, it may be compelled to separate the dyad, offering, a new, even if painful, way of life for children. In other cases, when there is a total closure toward the therapy of mothers, who boycott frequently therapeutic moments and interventions, equipe is compelled to separate the dyad forcedly, given that there is not a premise for a change.

AAI Question: ok, what future would you like your daughter to have?

Answer: not like mine..no, indeed, I will do everything for… because she would have a life…happy. Not like my mom said to me, “you never know it will make you freak out, that she would become like you who drove me crazy” she told me. And I said “I will not be the same mum as you were so she will not have reasons to do that.” (Silence) because she may hurt me whenever she wants… I always have to shut up because I am scared to hurt her… no, stop, this story is over. I’m tired… because I didn’t call her: “ means that she does not love me if she didn’t call me” she said to my boyfriend. So, you’re fifteen, you talk as you would be seven, s, c’mon (Silence)…(grumble) ok

## DISCUSSION

This study aimed at being a first attempt to organize a clinical model in an attachment framework, as to interpret multi-level assessment of SDD and BPD mothers, hosted in Health Care Services and TCs with their children. The main purpose is, so, improving and planning effective clinical and much more personalized interventions, in order to reduce risks of failure and useless therapeutic planning.

The present study deserves a reflection on the characteristics of the clinical population that follow a mandatory treatment in a residential mother–child TCs. The main purpose was to help clinicians and researchers to find more efficient models, in order to connect mother’s health with child’s well-being. This would allow to help both of them in the transition to foster care services and adoptive pathways when mother’s relational and attachment diseases do not allow the possibility for them to stay together. All mothers are led to comprehend their difficulties in the parental functioning, but not all of them are able to solve and overtake their trauma and severe personality dysfunctions. Indeed, the majority of these mothers understands its limitations in parenting, learns how to handle them and applies new strategies of caring: in this sub-group we find women who, as outcomes of their therapeutic pathway, maintain the relationship with their children more or less autonomously (see *Warm* and *Cold profiles*). Another sub-group includes those mothers who are able to understand their limits, not completely overtaking them: here we find those who, in alliance with their therapists’ thoughts, build pathways of custody or adoption. Finally, the last sub-group, which is fortunately rather small, includes those women who, because of their severe pathology, can not understand deeply their parental dysfunctions. For this reason, their kids are separated in a forced way from their mothers, against the latter’s will (see *Hot* and *Unpredictable profiles*). Not all mothers who adhere to this program of intervention are able to take care of their children after the therapeutic pathway. We have seen how trauma, personality and attachment history represent central aspects in respect to the outcomes: for this reason we think they should be better and more deeply studied, in order to select dyads which could have more possibilities to benefit from intervention programs. In general, the complexity of these mothers’ characteristics demands for an integrated model of intervention, which should include both representational and behavioral aspects connected to the attachment and to the general psychopathological functioning. In fact, each of the therapeutic accesses points will have to consider, on a side, the individual dysfunctionalities and, on another one, the quality of the interactions and of the relationship with the child.

We showed how the attachment framework (Bowlby, 1969; Wallin, 2007) offers a powerful model in which the quality of relational and attachment patterns help definitively to define mothers parenting profile. Whenever possible, paying attention to mothers’ clinical diagnosis, evaluating deficits and resources in their personality structure (DSM-IV diagnosis in Axes I and II), level of psychiatric symptoms and maladjustment could help on planning centered parent–child rehabilitation program. Given that, our “4 profiles-mother model” could be considered as a qualitative clinical pilot framework to plan treatment and to foresee outcome.

In conclusion, we hope that future research will employ larger samples as to validate our preliminary clinical findings.

The present paper presents a series of limitation that may provide new perspectives for future studies. The first limitation concerns the slenderness of the sample: 30 subjects do not constitute a representative group, and, for this reason, compromising the generalizability of results, even considering the homogeneity of the clinical population studied in our sample. Such small numbers do not allow neither for consistent statistical analysis about the influence of socio-demographic aspects on observed clinical and therapeutic characteristics, nor for the definition of an exhaustive and interpretative model of the connection between SDD, BPD, and distribution of attachment patterns, as evaluated by AAI.

Moreover, as for the applied methodology, two main points emerged. Firstly, SCL-90-R is a self-report measure that, when administered in mandatory conditions, could be influenced by social desirability. The possibility of adding another tool in which social desirability issues are taken into consideration could help in reducing the risk of obtaining biased scores on psychiatric scales (e.g., Millon Clinical Multiaxial Inventory-III, MCMI-III; Millon, 1983, 1997; Millon et al., 2008; Zennaro et al., 2008). Then, as regards the evaluation of mental representations linked to attachment, we consider that the AAI needs to be sustained with other tools, such as the Adult Attachment Projective Picture System (AAP; George and West, 2001). AAP specifically could allow for a deeper analysis of the disorganized attachment factors, the entity of traumatic experience and defensive mechanisms; these aspects could be highly useful given the complexity of these comorbid patients’ traumatic early experiences. Last but not least, it would be useful to introduce some quantitative and multiple-informant evaluation of parenting style and efficacy, in order to investigate both women’s perceptions of their skills in taking care of their children (e.g., using self-report questionnaires), and quality of real behaviors via observational measures (e.g., the Biringen Scales, EAS – Emotional Availability Scales; Biringen et al., unpublished manuscript).

## Conflict of Interest Statement

The authors declare that the research was conducted in the absence of any commercial or financial relationships that could be construed as a potential conflict of interest.

## Footnotes

1 The children were 14 females and 16 males, from newborn to 7 years old.

2 The access in the community for addicted mothers and their children is applied by a decree of the Juvenile Court and it implies a coercive intervention for the mother; the risk is to be immediately separated from her child (Stevens et al., 1989; National Institute on Drug Abuse, 1996), unless she accepts treatment programs offered.
